# Accuracy of hemodynamic parameters derived by GE E-PiCCO in comparison with PiCCO® in patients admitted to the intensive care unit

**DOI:** 10.1038/s41598-023-34141-8

**Published:** 2023-04-26

**Authors:** Katarzyna Putko, Johanna Erber, Franziska Wagner, Daniel Busch, Hannah Schuster, Roland M. Schmid, Tobias Lahmer, Sebastian Rasch

**Affiliations:** Department of Internal Medicine II, School of Medicine, University Hospital rechts der Isar, Technical University of Munich, Munich, Germany

**Keywords:** Medical research, Biophysics

## Abstract

To evaluate the agreement and accuracy of a novel advanced hemodynamic monitoring (AHM) device, the GE E-PiCCO module, with the well-established PiCCO® device in intensive care patients using pulse contour analysis (PCA) and transpulmonary thermodilution (TPTD). A total of 108 measurements were performed in 15 patients with AHM. Each of the 27 measurement sequences (one to four per patient) consisted of a femoral and a jugular indicator injection via central venous catheters (CVC) and measurement using both PiCCO (PiCCO® Jug and Fem) and GE E-PiCCO (GE E-PiCCO Jug and Fem) devices. For statistical analysis, Bland–Altman plots were used to compare the estimated values derived from both devices. The cardiac index measured via PCA (CIpc) and TPTD (CItd) was the only parameter that fulfilled all a priori-defined criteria based on bias and the limits of agreement (LoA) by the Bland–Altman method as well as the percentage error by Critchley and Critchley for all three comparison pairs (GE E-PiCCO Jug vs. PiCCO® Jug, GE E-PiCCO Fem vs. PiCCO® Fem, and GE E-PiCCO Fem vs. GE E-PiCCO Jug), while the GE E-PiCCO did not accurately estimate EVLWI, SVRI, SVV, and PPV values measured via the jugular and femoral CVC compared with values assessed by PiCCO®. Consequently, measurement discrepancy should be considered on evaluation and interpretation of the hemodynamic status of patients admitted to the ICU when using the GE E-PiCCO module instead of the PiCCO® device.

## Introduction

Advanced hemodynamic monitoring (AHM) is an important element in the management of critically ill patients admitted to the intensive care unit (ICU) as more detailed information regarding the cardiopulmonary and cardiovascular status is obtained. AHM is also used to differentiate causes of hemodynamic instability. Furthermore, adequate fluid replacement or vasoactive drug dosage can be controlled by AHM^1–8^.

Owing to the increased importance of AHM, several new and less-invasive monitoring techniques have been introduced into clinical practice over the past years^9,10^. To optimize patient outcomes, the acquired data should be as precise as possible^11,12^. With an increasing availability of different monitoring devices, the accuracy and precision of the measured values should be clarified, and the agreement between these devices remains to be evaluated.

Recently, another commercially available device for AHM, the GE E-PiCCO module (GE Healthcare, Helsinki, Finland), has been introduced into practice. Similar to the established PiCCO®-system (PULSION Medical Systems SE, Feldkirchen, Deutschland/Getinge AB, Solna, Schweden), measurements with the GE E-PiCCO are based on pulse contour analysis (PCA) and transpulmonary thermodilution (TPTD). PiCCO® is officially approved for the jugular and femoral CVC. To adjust measurements for femoral indicator injections an integrated correction equation is used. According to the manufacturer, GE E-PiCCO only allows proper measurements using jugular TPTD indicator injections.

Objective of this study is, to evaluate the accuracy of the GE E-PiCCO against the PiCCO® device. In addition, we analyze the accuracy of the GE E-PiCCO device for femoral indicator injection comparing measurements from femoral TPTD with jugular TPTD.

For that reason, we also evaluated.

## Materials and methods

The study has a cross over design in which measurements with the GE E-PiCCO and with the PiCCO as reference device are performed in every patient.

### Patients

Between October 2019 and January 2020, 15 patients admitted at a medical ICU of a German university hospital (Klinikum rechts der Isar, Technical University of Munich) were enrolled in this study. The key inclusion criterion was the presence of both a femoral and a jugular central venous catheter (CVC), for clinical reasons unrelated to the study. The leading reason for concomitant femoral and jugular CVC was hemodialysis treatment with a three-lumen Shaldon catheter. To evaluate the accuracy of stroke volume variation (SVV) and pulse pressure variation (PPV), pressure-controlled ventilated patients with sinus rhythm were considered only as spontaneous breathing or pressure-supported ventilation, and arrhythmia can interfere with the correct measurement of SVV and PPV^8,13,14^. Six of the 15 included patients met these criteria.

### Hemodynamic measurements

Each measurement sequence contained four consecutive TPTDs using PiCCO® via jugular access (PiCCO Jug), PiCCO® via femoral access (PiCCO Fem), GE E-PiCCO via jugular access (GE E-PiCCO Jug), and GE E-PiCCO via femoral access (GE E-PiCCO Fem). Each TPTD measurement was performed in triplicates, injecting 15 ml of < 8 °C cold 0.9% saline as a bolus. Bolus injections were manually administered and not coordinated with the respiratory cycle. Immediately after each TPTD, the following hemodynamic parameters were recorded:

- *PCA* Cardiac Index (CIpc), systemic vascular resistance index (SVRI), SVV and PPV.
- *TPTD* Cardiac Index (CItd), global end-diastolic volume index (GEDVI) and extravascular lung water index (EVLWI).

Body position, fluid status, catecholamine doses, and ventilator settings remained unchanged throughout a measurement sequence.

TPTD indicator injections were performed using five-lumen CVC (Arrow International, Inc., Subsidiary of Teleflex Incorporated, Reading, PA, USA) with a maximum intravascular length of 20 cm and a diameter of 9.5 Fr. or via a high-flow three-lumen Shaldon catheter (Achim Schulz-Lauterbach VMP GmbH, Iserlohn, Deutschland/JO-LINE GmbH & Co. KG, Hechingen, Deutschland) with a maximum intravascular length of 15 cm and a diameter of 18 G.

For PCA, a thermistor tipped arterial catheter (PULSIONS Medical Systems SE/Getinge AB) with a length of 20 cm and a diameter of 5 Fr. placed in the femoral artery or with a length of 22 cm and a diameter of 4 Fr. placed in the brachial artery were used. PulsioFlex or PiCCO-2-monitor (PULSION Medical Systems SE/Getinge AB) equipped with the most recent algorithm (V3.1 algorithm) were used for hemodynamic monitoring. The GE E-PiCCO module required the CARESCAPE™ B650 monitor (GE Healthcare) for hemodynamic monitoring.

### Statistical analysis

Data were tested via Shapiro–Wilk test for normal distribution. Results of quantitative characteristics are expressed as mean (± standard deviation [SD]) if normally distributed or median (interquartile range) if not normally distributed. Results of categorical characteristics are expressed as absolute and relative frequency.

To illustrate the agreement between the estimated parameters derived by PiCCO® and GE E-PiCCO from femoral and jugular CVC sites, Bland–Altman plots were used^15–17^ as neither correlation nor regression analysis is appropriate to evaluate the comparability between monitoring devices^18^. In this context, the agreement was validated by calculating the systematic error (bias) with 95% limits of agreement (LoA) as bias ± 2 SD. Additionally, the 95% confidence interval (CI) for the bias and LoA and the percentage error were calculated as published by Critchley and Critchley^19^. Clinically satisfactory boundaries for maximum acceptable differences of the bias and LoA were defined in advance (Online Resource 1).

All statistical analyses were performed using the MedCalc® Statistical Software Version 20.106 (Ostend, Belgium).

### Ethics approval and consent to participate

This study was performed in line with the principles of the Declaration of Helsinki. Approval was granted by the Ethics Committee of the Technical University of Munich (No 3049/11s). Informed consent was obtained from all participants included in the study or their legal representatives.

## Results

### Patient characteristics

A total of 15 patients with 27 measurement sequences (one to four sequences per patient) were included in this study resulting in 108 thermodilutions, which were used to analyze CIpc, CItd, GEDVI, EVLWI, and SVRI. SVV and PPV data could be analyzed from results of 10 measurement sequences performed in six patients on pressure-controlled ventilation and in sinus rhythm. Patient characteristics and demographic data are shown in Table 1.

**Table 1:** Patient characteristics and demographic data.

Patient characteristics and demographic data	All patients n = 15	Pressure-controlled ventilated patients with sinus rhythm n = 6
Sex, n	♀: 7 (46.7%)	♀: 3 (50.0%)
	♂: 8 (53.3%)	♂: 3 (50.0%)
Age, years	64.3 ± 15.0	59.8 ± 15.8
Height, cm	172.3 ± 6.9	172.8 ± 7.3
Weight, kg	81.6 ± 16.0	79.2 ± 13.6
Body mass index, kg/m^2^	27.5 ± 5.5	24.6 (24.5/26.6)
Body surface area (PiCCO), m^2^	1.9 (1.8/2.1)	2.0 ± 0.2
Predicted body surface area (PiCCO), m^2^	1.8 ± 0.2	1.8 ± 0.2
Predicted body weight (PiCCO), kg	65.4 ± 8.9	65.0 ± 9.0
Body surface area (GE E-PiCCO), m^2^	1.9 ± 0.2	1.9 ± 0.1
SOFA score	8.8 ± 3.4	11.2 ± 2.7
APACHE II score	18.8 ± 8.1	19.5 ± 8.2
Reason for ICU admission		
Sepsis	2 (13.3%)	0
Liver diseases	2 (13.3%)	1 (16.7%)
Pulmonary diseases	3 (20.0%)	2 (33.3%)
Cardiogenic diseases	2 (13.3%)	0
Gastrointestinal diseases	3 (13.3%)	1 (16.7%)
Others	3 (20.0%)	2 (33.3%)

Quantitative characteristics: normally distributed: mean ± SD; not normally distributed: median (interquartile range).

Categorical characteristics: an absolute (relative) frequency.

*APACHE II Score* acute physiology and chronic health evaluation II score, *SOFA Score* sequential organ failure assessment score.

Table 2 shows the cardiopulmonary characteristics and vital parameters at the beginning of each measurement sequence. At the time of measurement 14 patients were in shock with the need of vasopressor therapy. Eleven of these patients received noradrenaline, 1 patient noradrenaline plus dopamine and 1 patient terlipressin. One patient was in acute respiratory distress syndrome. Relevant cardiovascular co-morbidities were present in 4 patients (2 × coronary heart disease, 2 × aortic aneurysm repair).

**Table 2:** Cardiopulmonary characteristics and vital parameters.

Cardiopulmonary characteristics and vital parameters	All measurement sequences n = 27	Measurement sequences in pressure-controlled ventilated patients with sinus rhythm n = 10
Mechanical ventilation		
Pressure supported	11 (40.7%)	
Pressure controlled	10 (37.0%)	10 (100%)
Vt/PBW [ml/kg]	6.3 (4.6/17.2)	6.3 ± 1.0
PEEP	8 (6/10)	8 (6/10)
Spontaneous breathing	6 (22.5%)	
Breathing rate, 1/min	20.0 ± 7.3	22 (20/24)
Oxygen saturation, %	95.2 ± 2.5	95.5 ± 2.1
P/F ratio (mmHg)	256 ± 62	243 ± 62
PVPI > 3.5	2 (7.0%)	
Mean arterial pressure, mmHg	82.2 ± 13.6	88.3 ± 11.8
Heart rate, 1/min	80.1 ± 19.5	83.0 ± 20.0
Sinus rhythm	22 (81.5%)	10 (100.0%)
Vasopressor therapy	14 (51.9%)	6 (60.0%)
Noradrenaline dose (µg/kg/min)	0.03 (0.01/0.15)	0.08 (0.02/0.15)
ScvO2 (%)	72.9 ± 9.6	
Core body temperature, °C	36.3 ± 0.8	35.9 ± 0.9
Central venous pressure (Jug), cmH_2_O	16.1 ± 7.4	19.3 ± 7.2
Arterial pH	7.4 ± 0.1	7.4 ± 0.1
Glasgow coma scale	3 (3/11)	3 (3/3)

Quantitative characteristics: normally distributed: mean ± SD; nonnormally Distributed characteristics: median (interquartile range).

Categorical characteristics: absolute (relative) frequency, *Vt* tidal volume, *PBW* predicted body weight, *PEEP* positive endexpiratory pressure, *P/F ratio* Horovitz index, *PVPI* pulmonary vascular permeability index, *ScvO2* central venous oxygen saturation.

### Comparison between GE E-PiCCO Jug and PiCCO® Jug

Table 3 shows the mean values (± SD) for the investigated hemodynamic parameters CIpc, CItd, GEDVI, EVLWI, SVRI, SVV, and PPV.

**Table 3:** Mean values with SD for CIpc, CItd, GEDVI, EVLWI, SVRI, SVV, and PPV for each device (Jug/Fem).

Mean values of the investigated parameters	GE E-PiCCO Jug Mean ± SD	GE E-PiCCO Fem Mean ± SD	PiCCO® Jug Mean ± SD	PiCCO® Fem Mean ± SD
CIpc, l/min/m^2^ n = 27	3.57 ± 0.69	3.85 ± 0.77	3.67 ± 0.78	3.82 ± 0.75
CItd, l/min/m^2^ n = 27	3.52 ± 0.70	3.80 ± 0.75	3.64 ± 0.75	3.83 ± 0.71
GEDVI, ml/m^2^ n = 27	811.1 ± 169.1	1067.3 ± 262.3	849.0 ± 194.3	811.7 ± 171.4
EVLWI, ml/kg n = 27	9.0 ± 2.8	10.1 ± 3.3	9.2 ± 3.1	10.3 ± 3.0
SVRI, dyn⋅s⋅cm^−5^⋅m^2^ n = 27	1617.1 ± 510.7	1454.4 ± 483.4	1535.8 ± 490.7	1443.0 ± 529.4
SVV, % n = 10	6.2 ± 5.4	8.2 ± 8.7	8.1 ± 7.6	7.9 ± 9.5
PPV, % n = 10	6.1 ± 7.2	7.1 ± 8.7	6.9 ± 7.2	7.0 ± 7.6

We first compared the results for the parameters CIpc, CItd, GEDVI, EVLWI, SVRI (n = 27) and SVV, PPV (n = 10) assessed with the GE E-PiCCO and PiCCO® devices upon jugular injection using Bland–Altman analysis (Table 4).

**Table 4:** Comparison between GE E-PiCCO Jug and PiCCO® Jug: CIpc, CItd, GEDVI, EVLWI, and SVRI (n = 27).

GE E-Picco jug Vs. Picco® jug	CIpc, l/min/m^2^	CItd, l/min/m^2^	GEDVI, ml/m^2^	EVLWI, ml/kg	SVRI, dyn⋅s⋅cm^-5^⋅m^2^	SVV, %	PPV, %
Bias [95% CI]	− 0.11 [− 0.22; 0.01]	− 0.12 [− 0.22; − 0.03]	− 37.9 [− 56.4; − 19.4]	− 0.1 [− 0.4; 0.1]	81.4 [2.1; 160.7]	− 1.9 [4.0; 0.2]	− 0.8 [− 1.7; 0.1]
SD_Dif_ × 1.96	0.57	0.46	91.8	1.4	392.9	5.9	2.4
Upper LoA [95% CI]	0.46 [0.26; 0.66]	0.33 [0.17; 0.49]	53.9 [21.9; 86.0]	1.3 [0.8; 1.8]	474.2 [337.0; 611.5]	4.0 [0.2; 7.8]	1.6 [0.1; 3.2]
Lower LoA [95% CI]	− 0.67 [− 0.87; − 0.48]	− 0.58 [− 0.74; − 0.42]	− 129.7 [− 161.8; − 97.6]	− 1.6 [− 2.0; − 1.1]	− 311.5 [− 448.7; − 174.3]	− 7.8 [− 11.6; − 4.0]	− 3.2 [− 4.8; − 1.7]
PE, %	15.6%	12.7%	11.1%	15.4%	24.9%	81.7%	36.2%

*SD*_*Dif*_ standard deviation of the difference between the methods, *Upper LoA* upper limits of agreement (bias + (1.96 × SD_Dif_)), *lower LoA* lower limits of agreement (bias − (1.96 × SD_Dif_)), *PE* percentage error.

### Comparison between GE E-PiCCO Fem and PiCCO® Fem

Next, results between GE E-PiCCO Fem and PiCCO® Fem were compared for the parameters CIpc, CItd, GEDVI, EVLWI, SVRI (n = 27) and SVV, PPV (n = 10) (Table 5).

**Table 5:** Comparison between GE E-PiCCO Fem and PiCCO® Fem.

GE E-PiCCO Fem vs. PiCCO® Fem	CIpc, l/min/m^2^	CItd, l/min/m^2^	GEDVI, ml/m^2^	EVLWI, ml/kg	SVRI, dyn⋅s⋅cm^−5^⋅m^2^	SVV, %	PPV, %
Bias [95% CI]	0.03 [− 0.12; 0.17]	− 0.02 [− 0.13; 0.08]	255.6 [208.8; 302.3]	− 0.1 [− 0.5; 0.3]	11.4 [− 85.3; 108.1]	0.3 [− 0.8; 1.4]	0.1 [− 1.6; 1.8]
SD_Dif_ × 1.96	0.70	0.53	231.6	1.8	479.3	3.1	4.5
Upper LoA [95% CI]	0.72 [0.48; 0.97]	0.51 [0.32; 0.69]	487.2 [406.3; 568.1]	1.7 [1.1; 2.4]	490.7 [323.3; 658.1]	3.4 [1.4; 5.4]	4.7 [1.7; 7.6]
Lower LoA [95% CI]	− 0.67 [− 0.92; − 0.43]	− 0.55 [− 0.74; − 0.37]	24.0 [− 56.9; 104.8]	1.9 [− 2.6; − 1.3]	− 46.9 [− 635.2; − 300.5]	− 2.8 [− 4.8; − 0.8]	− 4.5 [− 7.4; − 1.5]
PE, %	18.2%	13.9%	24.7%	17.9%	33.1%	38.7%	63.5%

*SD*_*Dif*_ standard deviation of the difference between the methods, *Upper LoA* upper limits of agreement (bias + (1.96 × SD_Dif_)), *lower LoA* lower limits of agreement (bias − (1.96 × SD_Dif_)), *PE* percentage error.

### Comparison between GE E-PiCCO Fem and GE E-PiCCO Jug

Finally, GE E-PiCCO Fem and GE E-PiCCO Jug results for the parameters CIpc, CItd, GEDVI, EVLWI, and SVRI (n = 27) and for SVV and PPV (n = 10) were compared (Table 6).

**Table 6:** Comparison between GE E-PiCCO Jug and GE E-PiCCO Fem.

GE E-PiCCO Jug vs. GE E-PiCCO Fem	Cipc, l/min/m^2^	Citd, l/min/m^2^	GEDVI, ml/m^2^	EVLWI, ml/kg	SVRI, dyn⋅s⋅cm^−5^⋅m^2^	SVV, %	PPV, %
Bias [95% CI]	0.28 [0.13; 0.43]	0.29 [0.13; 0.45]	256.1 [194.1; 318.1]	1.1 [0.4; 1.8]	− 162.8 [− 234.1; − 91.5]	2.0 [− 1.0; 5.0]	1.0 [− 0.5; 2.5]
SD_Dif_ × 1.96	0.75	0.79	307.1	3.7	353.4	8.0	4.1
Upper LoA [95% CI]	1.04 [0.77; 1.30]	1.07 [0.80; 1.35]	563.2 [456.0; 670.5]	4.8 [3.5; 6.0]	190.6 [67.2; 314.0]	10.1 [4.9; 15.3]	5.1 [2.5; 7.8]
Lower LoA [95% CI]	− 0.47 [− 0.73; − 0.21]	− 0.50 [− 0.77; − 0.22]	− 51.0 [− 158.3; 56.3]	− 2.5 [− 3.8; − 1.3]	− 516.1 [− 639.6; − 392.7]	− 6.1 [− 11.3; − 0.9]	− 3.1 [− 5.8; − 0.5]
PE, %	20.3%	21.5%	32.7%	38.2%	23.0%	111.6%	62.4%

*SD*_*Dif*_ standard deviation of the difference between the methods, *Upper LoA* upper limits of agreement (bias + (1.96 × SD_Dif_)), *lower LoA* lower limits of agreement (bias − (1.96 × SD_Dif_)), *PE* percentage error.

### Evaluation of the results for clinical acceptance

**Figure 1:**
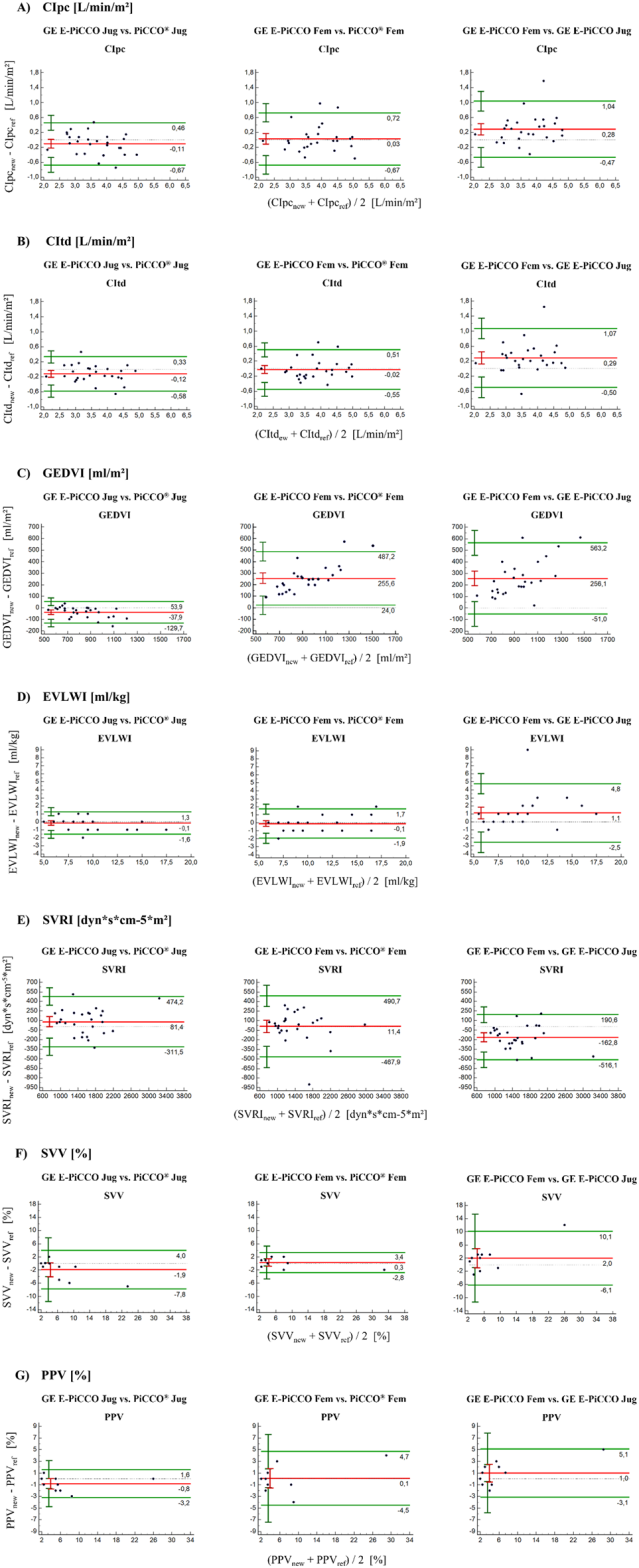
Bland–Altman plots. The points are based on means of each injection method and each AHM device. The red line represents the bias. The green lines indicate the upper and lower LoAs. The vertical bars represent the 95% CI.

Figure 1 shows Bland–Altman plots for the hemodynamic parameters of each CVC position. The bias of CIpc, CItd, and PPV measurements was within the a priori-defined criteria (Online Resource 1) for all three comparisons (GE E-PiCCO Jug vs. PiCCO® Jug, GE E-PiCCO Fem vs. PiCCO® Fem, and GE E-PiCCO Fem vs. GE E-PiCCO Jug, Fig. 1A,B,G). Further, the bias of EVLWI and SVRI was within the predefined limits for jugular and femoral TPTD (Fig. 1D,E). The SVV was within the set limits when comparing GE E-PiCCO Fem and PiCCO® Fem (Fig. 1F). The GEDVI was within the predefined limits when comparing GE E-PiCCO Jug and PiCCO® Jug (Fig. 1C). However, comparing femoral and jugular injection of the indicator, a trend to an overestimated GEDVI was identified by the GE E-PiCCO compared to the PiCCO® device: With higher GEDVI values, an increasing inaccuracy of the GE E-PiCCO was observed (Fig. 1C).

Only CIpc and CItd measurements in all three comparisons and GEDVI measurements in the comparison between GE E-PiCCO Jug and PiCCO® Jug were within a priori-defined range (Online Resource 1) of the LoA using the Bland–Altman analysis. LoA values of the remaining hemodynamic parameters did not fulfill the limits set for measurement precision.

A PE of ≤ 30% has been suggested as an acceptable correlation between two devices by Critchley and Critchley^19^. For both CIpc and CItd, PEs remained to be < 30% in all three comparisons. For GEDVI and EVLWI, PEs of the comparisons GE E-PiCCO Jug vs. PiCCO® Jug and GE E-PiCCO Fem vs. PiCCO® Fem were < 30%. For SVRI, PEs of comparisons GE E-PiCCO Jug vs. PiCCO® Jug and GE E-PiCCO Fem vs. GE E-PiCCO Jug were < 30%. None of the PE values calculated for SVV and PPV were within the 30% PE cutoff.

## Discussion

This study compares the accuracy and conformity between two AHM devices, the novel GE E-PiCCO module and the established PiCCO® system. AHM is considered to be an important element in the diagnostic and therapeutic management of critically ill patients admitted to the ICU^20^. The hemodynamic status has a major impact on patients’ therapy. Thus, for an optimal patient outcome, a proper data interpretation of AHM is mandatory, which in turn relies on the acquisition of accurate and precise hemodynamic parameters^11,12^.

Using the LoA method, we show that measurements of CI (CItd and CIpc) agree between the GE E-PiCCO module and the PiCCO® device regardless the position of the CVC. However, our study results indicate that the hemodynamic parameters EVLWI, SVRI, SVV, and PPV assessed with the GE E-PiCCO module deviate from the measurements of the PiCCO® device for jugular and femoral indicator injection. Regarding GEDVI, results differ between the two devices after femoral indicator injection.

### Effects of CVC site on parameters measured by TPTD

Several studies demonstrated a GEDVI overestimation in case of TPTD indicator injections through a femoral instead of a jugular or subclavian CVC^21–23^, which can be explained by the higher mean transit time (MTt) due to the increased distance of the tip of the femoral CVC to the right atrium. Consequently, the additional volume in the inferior vena cava extends the MTt^21,23^. As the MTt is needed to calculate GEDVI and EVLWI, MTt changes also result in GEDVI and EVLWI changes^21^. For that reason, a correction formula for GEDVI measured with TPTD using femoral indicator injection has been suggested^22^.

The PiCCO® device demands information about the CVC site (jugular or femoral) to correct specific parameters. In contrast, the GE E-PiCCO device does not request information about the applied CVC position. Consistent with the results of Schmidt et al., Saugel et al., and Huber et al., this study also shows that GEDVI is overestimated in case of femoral indicator injection and use of the GE E-PiCCO device^21–23^, suggesting that an application of a correction formula for the femoral CVC site for the GE E-PiCCO module, as suggested by Saugel et al., could reduce the measurement inaccuracy of GEDVI and possibly of EVLWI using the GE E-PiCCO device^22^.

### Measurement inaccuracy of hemodynamic parameters derived via PCA

The hemodynamic parameters CIpc, SVRI, SVV, and PPV can be continuously derived via PCA. Our data show that GE E-PiCCO reliably estimates the CIpc via PCA, whereas agreement with the PiCCO® device is lower for SVRI, SVV, and PPV.

SVV and PPV are dynamic variables used to predict fluid responsiveness in mechanically, pressure-controlled ventilated patients^14,24,25^. For this purpose, SVV and PPV are derived by the analysis of dynamic changes in the arterial waveform.

In this study, four consecutive TPTDs were performed to calibrate the PCA for femoral and jugular injection, assessed with the two devices. Each TPTD required the application of a minimum of three indicator injections of 15 ml cold saline. Consequently, at least 180 ml were administered intravenously, increasing the intravascular volume during the measurement sequence. Physiologically, an increase in intravascular volume results in increased venous return and stroke volume. In line with this, Biais et al. demonstrated that a mini-fluid challenge of 100 ml can lead to changes in stroke volume index and PPV^26^. In contrast, Aya et al. reported that a fluid volume of 321 to 509 ml is required for an effective fluid challenge resulting in changes of the cardiac output and mean systemic filling pressure, which significantly exceeds the fluid volume administered during the measurement sequence of this study protocol^27^. Additionally, Toscani et al. reported that a fluid infusion time of more than 30 min was not as effective as a fluid bolus administered in less than 30 min^28^. With a slower infusion rate, the increase of venous return and stroke volume is lower as opposed to a rapidly administered fluid bolus^28^. In this study, one measurement sequence comprising four TPTDs took longer than 30 min. We cannot exclude that the increase in preload resulting from the fluid bolus applied in this study might have affected the hemodynamic status and consequently the SVV and PPV results over the course of a measurement sequence. Given the fluid volume of 180 ml administered over more than 30 min, we consider significant changes to the hemodynamic situation to be unlikely.

The SVRI has hardly ever been analyzed or discussed in studies comparing two AHM devices. However, the SRVI is a hemodynamic parameter that is not directly measured but computed, dividing the difference of the mean arterial pressure (MAP) and central venous pressure using CI, multiplied by the constant 80^29^. Both, the CI and the MAP are dynamic variables that may change with every heartbeat, offering an explanation for differences in the SVRI during the measurement sequence, although “beat-to-beat” alterations of CI or MAP are considered to be minimal.

### Study limitations

As patients with both jugular and femoral venous accesses were required, this study is limited by its rather small patient cohort and might consequently be subject to a greater measurement uncertainty^30,31^. Further, we could not attribute divergent measurements to differences in the applied algorithms as we had no access to information regarding the computational details of both AHM devices. The GE E-PiCCO device was compared with the PiCCO® system as a reference. Although the latter device has been repeatedly demonstrated to be a reliable AHM device^32^, it is only one approach to reflect the complexity of a patient’s hemodynamic situation. Therefore, the study design only allows analysis of the performance of the GE-PiCCO system in comparison with the established PiCCO® device, while it remains uncertain which of the two devices ascertains the physiological values more accurately.

## Conclusion

In patients admitted to the ICU, the GE E-PiCCO module generates divergent values for the parameters EVLWI, SVRI, SVV, and PPV via jugular as well as femoral CVC, compared to the PiCCO® device. Regarding GEDVI, the GE E-PiCCO device only achieved a satisfactory agreement with the PiCCO® for results via a jugular CVC. However, for parameters CIpc and CItd, the GE E-PiCCO device showed a good agreement with the PiCCO® as a reference method via jugular and femoral CVC.

In conclusion, it is important to know that values measured with either of the GE E-PiCCO or PiCCO® system are not necessarily transferrable to the other which might affect the interpretation of the hemodynamic status and consequently therapeutic decisions. In particular, the lack of a correction for femoral CVC placement limits the use of GE-E-PiCCO in patients with femoral venous access to date.

## Supplementary Information

Supplementary Information.

## Data Availability

The datasets analysed during the current study are available from the corresponding author on reasonable request.
