# Supplementary material for: Accuracy of hemodynamic parameters derived by GE E-PiCCO in comparison with PiCCO® in patients admitted to the intensive care unit
Source: Sci Rep. 2023 Apr 26;13:6861. doi: 10.1038/s41598-023-34141-8 (PMC10133386; doi:10.1038/s41598-023-34141-8)
Supplement: Supplementary file 1 — Supplementary Information. [file 41598_2023_34141_MOESM1_ESM.docx]

**Online Resource 1: A priori-defined criteria for clinical acceptance**

|  | **Bias** | **LoA** |
| --- | --- | --- |
| **CIpc, l/min/m²** | −0.50 ≤ Bias ≤ 0.50 | Bias ± 1.00 |
| **CItd, l/min/m²** | −0.50 ≤ Bias ≤ 0.50 | Bias ± 1.00 |
| **GEDVI, ml/m²** | −150.0 ≤ Bias ≤ 150.0 | Bias ± 150.0 |
| **EVLWI, ml/kg** | −1.0 ≤ Bias ≤ 1.0 | Bias ± 1.0 |
| **SVRI, dyn*s*cm^-5^*m²** | −150.0 ≤ Bias ≤ 150.0 | Bias ± 150.0 |
| **SVV, %** | −1.0 ≤ Bias ≤ 1.0 | Bias ± 1.0 |
| **PPV, %** | −1.0 ≤ Bias ≤ 1.0 | Bias ± 1.0 |
